# Non-Hormonal Treatments for Vasomotor Symptoms of Menopause: An Up-to-Date Review

**DOI:** 10.7759/cureus.117084

**Published:** 2026-09-28

**Authors:** Ala'a S Farhan, Hetaf S Farhan, Mohammed T Dinfash

**Affiliations:** 1 Obstetrics and Gynecology, University of Anbar-College of Medicine, Ramadi-Al-Anbar, IRQ; 2 Biology, Directorate of Anbar Agriculture, Al-Anbar, IRQ; 3 Pharmacology, Amiriyah General Hospital, Amiriyah, IRQ

**Keywords:** black cohosh, fezolinetant, menopause, non-hormonal, vasomotor symptoms

## Abstract

Treatment of vasomotor symptoms, sweating, and hot flashes after menopause is important to improve the quality of life in this population because of the burden and disruption that these features exert on daily life activities. In recent times, there has been a focus on non-hormonal modalities for the treatment of vasomotor symptoms because of the elevated concern about the adverse effects of hormonal treatment and the presence of contraindications to its use in some patients. Therefore, our objective in this review is to focus on non-hormonal options and their efficacy and effectiveness in managing vasomotor symptoms.

To improve the quality of life for women going through menopause, this review summarizes recent research on menopausal vasomotor symptoms, including the physiology and pathophysiology of menopause and most non-hormonal methods for treating these symptoms. Improving life quality in menopausal women by relieving the troublesome vasomotor symptoms during this period of life is essential, so searching for new modalities of treatment that are safe and well tolerated is important. There are many non-hormonal options for treating the vasomotor symptoms in menopause, with variable effects and potency as well as fewer side effects and contraindications than hormonal types. These options include non-drug-like psychotherapies, herbal supplements, and drugs that have relative safety and more tolerability than hormonal options.

## Introduction and background

The transformation from procreative to post-procreative life in women is called perimenopause, and it indicates an important landmark in a woman's lifetime [1]. It occurs around one year after the final menstrual cycle, which ceases due to a progressive decline in ovarian activity [2].

Menopause is associated with a lot of physical, mental, and genitourinary symptoms that may negatively influence general well-being. Sociodemographic, gynecologic, and cultural traits influence symptom intensity [3]. Moreover, research concentrated on women in Indian rural areas denoted that women in perimenopause are more likely to suffer symptoms of menopause in comparison to women in pre-menopause and post-menopause [4].

Vasomotor features, called hot flashes and night sweats, are the characteristic features of transition to menopause. These features, defined as short 1-5-minute attacks of elevated heat and reddening in the upper part of the body, are usually associated with increased heart rate and anxiety, followed by a rapid decrease in internal body temperature and sweating [5]. Hot flash rate varies from infrequent attacks per week to one or two episodes per hour [6].

As many as 80% of women suffer vasomotor symptoms in menopause, and most women assess them as moderate to severe [7]. This happens in the setting of huge variations in the ultimate estradiol elimination in perimenopause [8]. Vasomotor symptoms are considered the most irritating feature due to their unintentional onset [9]. Unconventionally, they were considered temporary features of the menopause transition, but new research proposed that they can continue for more than seven years after menopause onset [10].

Its pathophysiology is not fully known but is thought to involve alterations in the thermoregulatory system, which maintains core body temperature within the thermoregulatory zone [11]. Vasomotor features may lead to sleep disturbance, and combined vasomotor features and defective sleep may interfere with daily routine, quality of life, and effectiveness at home and work. In one study, almost half of postmenopausal women experienced disturbed sleep. These were also related to inferior quality of sleep and excessive daytime sleepiness [12]. This is often the initial reason women seek medical care for symptoms associated with menopause [13].

In this narrative review, we focus on non-hormonal therapy for vasomotor features of menopause because of its relative safety and fewer side effects than hormonal types of treatment. We collected up-to-date information on vasomotor symptoms of menopause from several international publishers by reviewing and summarizing recent literature on menopause, the pathophysiology of vasomotor symptoms, and previous and current treatment options, given the burden of these symptoms on menopausal women.

## Review

Transition to menopause

Menopause is a natural progression leading to the termination of women’s reproductive ability, described by the irreversible cessation of menstruation, stoppage, and decrease in the functional role of the ovaries. In addition to its generative consequences, menopause has been accompanied by a decrease in well-being across many body systems, including an elevated risk of cardiovascular, mental, and musculoskeletal conditions [14]. Nowadays, approximately 95% of women in many societies are in the menopause period, and this population is expected to reach 1.2 billion by 2030, with approximately 47 million women entering menopause each year [15].

Menopause ideally happens between 45 and 55 years of age in women. The transition to menopause is classified into three stages: climacteric change, menopause, and postmenopausal phase. When menopause occurs before 45 years, it is categorized as premature menopause, while menopause that happens below the age of 40 years is known as premature ovarian failure [16]. The shift to menopause is a healthcare turning point for women, affecting factors that extend far beyond the generation. In addition to the clinical features that occur with menopause, associated organic, psychological, mental, and social changes affect middle-aged women and their well-being in the years to come [17].

The transition to menopause begins with the initiation of irregularities in the menstrual cycle and continues until the final menstruation. This change is described as a steady change from perimenopause to menopause and points to one of the most important phases of middle-aged women's life [18]. Initially, during the transition to menopause, menstruation is normal, yet menstrual cycle duration varies from normal. Ordinarily, menstrual cycles become shorter as a result of follicular phase shortening. Because of a reduction in inhibin levels, follicle-stimulating hormone (FSH) levels increase because of the absence of the negative impact of inhibin. As a consequence, levels of estradiol can also be temporarily elevated [19]. The site of residency, race, teaching level, and mode of life may affect the frequency of menopausal symptoms [20].

Clinical features of menopause

The clinical features women experience as they reach the stages of the shift to menopause, specifically the last generative phase of childbearing age, are largely unknown [21]. The features related to the perimenopausal period fall into several categories. Vasomotor symptoms, such as night sweats or hot flashes, are traditional features associated with the perimenopausal period. Extra-somatic features can include urogenital complications such as incontinence of urine, dryness of the vagina, or reduction of libido. Sleep disruption is common, with increased insomnia and sleep difficulty, and is often related to vasomotor symptoms [22].

Public health information shows that there is an elevated risk of developing ischemic heart problems in menopausal women. The protective role of estrogen on the heart is due to its effects on lipoproteins and lipid metabolism, as well as its role as a chemical intermediary that affects the vasculature. Osteoporosis influences female health and life expectancy; in the coming years, the frequency of osteoporosis-associated elements will be strongly elevated [23].

Although they are not commonly stated, genitourinary features usually occur after menopause and consist of itching of the vulva, dryness of the vagina, stinging and dyspareunia, dysuria, urinary frequency and urgency, and repeated lower urinary tract infections [24]. Another important point about this feature is that, despite the high frequency of women complaining of urinary features, only a small proportion seek assistance because of these complaints. This suggests that women either do not recognize these as managing features of menopause or do not feel comfortable requesting assistance [25].

During menopause, depression is usually subtle, showing unusual symptoms that defy conventional diagnostic systems. As opposed to obvious sadness, women may complain of testiness, an inability to feel pleasure, or exhaustion, features that can be easily confused with usual ageing or tension. Depression at the start of the perimenopausal period indicates a distinctive cause linked to the hormonal change [26].

Estrogens play common neuroregulatory roles in myelin genesis, synaptic plasticity, neuroimmune state, and mitochondrial function, and testosterone and progesterone have also been involved in neuroprotection. Concurrently, these findings suggest that menopausal age might be an element in long-term mental flexibility and vulnerability to neuropathic alterations [27].

Despite extensive recognition of menopausal symptoms and their important impact, numerous women avoid requesting therapeutic advice or receive insufficient therapy because of inadequate understanding, worries regarding therapeutic modalities, or the belief that these symptoms should be tolerated. This can result in persistent symptoms and a decrease in quality of life [28].

Hormonal treatment for menopause

Hormonal treatment with estrogen alone or a combined estrogen and progesterone is the most potent treatment for menopausal vasomotor symptoms. However, it is estimated that only 10% of women worldwide with irritating menopausal symptoms utilize drug treatment in the form of hormonal therapy, despite the interruption of these symptoms affecting their quality of life [29].

For women who have hormonal therapy contraindications, or those who prefer to avoid hormones, non-hormone-related and supplementary choices may be selected. Up-to-date non-hormone-related choices are likely to be accessible in the future. The application of hormone therapy in medical practice has formed different controversial opinions according to the results of interventional studies. Health hazards related to its utilization have raised concerns [30].

Non-hormonal treatment for menopause

Recently, more than two-thirds of women seeking therapy for menopausal symptoms have not been managed with hormonal therapy [31]. The use of supplementary and alternative treatments for managing menopausal symptoms has increased over the past several decades, although worries about their safety and efficacy remain [32].

Lifestyle Modifications and Behavioral Intervention

The recent guidance includes diet, steady exercise, lifestyle alteration, yoga, massage therapy, and additional stress management techniques. Many therapeutic measures are available to date for the treatment of menopausal symptoms. Among all the accessible treatments, dietary supplements and yoga play an efficient role in treating menopausal symptoms because of their low cost and minimal requirement of energy and time [33].

One possible behavioral practice is weight reduction in treating hot flashes. However, the role that weight has in hot flashes during the transition to menopause has been a controversial issue. Because adipose tissue is a peripheral site for the conversion of androgens to estrogens, body fat was originally postulated as protective against hot flashes. Despite this, more recent epidemiological research contests this opinion, as cross-sectional studies have shown that women with elevated body mass index and body fat experience hot flashes more than their thinner colleagues [34].

Acupuncture has appeared as a supplementary method for insomnia in perimenopausal women [35]. Initial studies indicate a notable decrease of more than 50% in hot flushes in women managed with acupuncture or electroacupuncture [36].

A systematic, organized training plan is an effective way to manage vasomotor symptoms. Developing evidence demonstrates that organized exercise, consisting of aerobic and resistance management strategies, has both favorable and limited impact on decreasing menopausal symptoms. Results from research are still mixed, with a few studies showing a decrease in symptoms and others reporting no progress or even temporary deterioration through follow-up tasks [37].

Behavioral treatment includes tranquility and gradual, deep abdominal breathing, which has been shown to reduce the mild-to-moderate severity of hot flashes. These types of breathing exercises are an extremely essential part of any yoga exercise [38].

Herbal and Dietary Options

Diet has appeared as a mainstay of well-being in menopause treatment, offering evidence-based, non-drug options that may adjust inflammatory, hormonal, and metabolic processes. Foods fortified with anti-inflammatory and phytoestrogen-containing nutrients have been frequently connected to the reduction in the intensity of vasomotor symptoms and the preservation of bone structure [39].

A lot of women demonstrate a preference for natural medicine and dietary supplements for menopausal symptoms, including cannabis, soy products, black cohosh, ammonium succinate, rhubarb, and probiotics. However, confirmation of a diminution of vasomotor symptoms with these products is limited or variable. Recently, none of these can be proposed as an empirically supported treatment [40]. Black cohosh is an extensively investigated herbal medicine, a North American plant that has been used previously as traditional medicine for menopausal symptoms [41]. Vitamin E is well known for its anti-inflammatory actions, which are thought to be a mechanism for relieving vasomotor features [42].

The inclusion of nutrient alterations and stress-reduction practices recognizes the varied character of health and well-being. By addressing these particular aspects, the intervention provided a functional tool for treating common symptoms of menopause, such as temperature fluctuations, hot flashes, and sleep disruption [43].

Psychotherapeutic Options

Behavioral therapy, like cognitive behavioral treatment, has been suggested as effective for management of menopausal symptoms [44]. It has confirmed effectiveness in relieving vasomotor features, although these studies have utilized multiple equivalents (routine care, waiting, and other functional regulators) and outcome indicators, restricting comparisons with pharmaceutical therapy [45].

Cognitive behavioral therapy customized for menopause helps improve coping mechanisms for managing vasomotor symptoms, mood fluctuations, and sleep difficulties. Treatment addresses irrational beliefs associated with menopause (such as thinking that symptoms are unmanageable or disgraceful) and uses procedures such as relaxation exercises, cognitive reframing, and controlled respiration [46].

Mind-body training represents a safe type of aerobic exercise. Conventional Asian methods like Ba Duan Jin and Tai Chi are especially available for women in society or the domestic environment because they do not require any specific instrument and are easy to perform. These methods may give moderate gains for both somatic and psychological well-being, as well as enhancement in overall standard of living among women in the peri- and post-menopausal periods [47].

Pharmacological Methods

Selective serotonin reuptake inhibitors: Antidepressants could be a practical therapy choice for mood-associated features of menopause, specifically in women having hormonal treatment contraindications. However, there is still debate on whether antidepressants are excessive for decreasing menopausal-related psychological features [48].

Although not as effective as hormone replacement treatment, selective serotonin reuptake inhibitors are powerful in decreasing the repetition and severity of hot flashes in menopausal women. At the molecular level, estrogen helps regulate serotonergic signaling by increasing the concentration of serotonin receptors in specific structures and directly raising serum serotonin levels through control of tryptophan hydroxylase. Therefore, as estrogen generation decreases in menopause, serotonin levels also fall [49].

Selective serotonin reuptake inhibitors block serotonin reuptake and activate serotonin receptor subtypes 2A and 1A, increasing serotonin levels. This avoids hyperthermia and suppresses hypothermia, providing an effective method for decreasing hot flashes with selective serotonin reuptake inhibitors [50].

Gabapentin and pregabalin: Gabapentin and pregabalin are anticonvulsant drugs capable of diminishing the repetition of hot flashes by binding to calcium channels situated in the hypothalamus and, accordingly, improving thermoregulatory function [51].

Clonidine: Antihypertensive drugs like clonidine, which is an alpha-adrenergic agonist, might be regarded as another non-hormonal choice for vasomotor symptoms and sleep disturbance. However, the effectiveness of clonidine therapy for recurrent hot flashes is moderate. Probable modes of action may include decreased levels of norepinephrine, which are increased as a result of the removal of estrogen and hot flashes [52].

Oxybutynin: Initially, oxybutynin was used for neurogenic bladder and is the most commonly prescribed medication for neurogenic bladder worldwide [53]. Women taking oxybutynin reported improvements in vasomotor symptoms, sleep, and quality of life at four weeks, with continued efficacy at 12 weeks [54]. Oxybutynin is an anticholinergic medicine approved by the US Food and Drug Administration as a therapy for overactive bladder features. Reduced sweating is a common side effect of oxybutynin, which supports its use in managing hyperhidrosis, and retrospective information showed that it might additionally be effective in treating recalcitrant hot flashes [55].

Fezolinetant: In recent years, a nonhormonal drug named fezolinetant, a nonhormonal neurokinin 3 receptor antagonist, has emerged for the treatment of moderate to severe vasomotor features [56]. It provokes neurokinin B, kisspeptin, neurokinin B, and dynorphin neurons located in the hypothalamus; fezolinetant reduces neurotransmission, resulting in a decrease in hot flashes [57].

Phytoestrogens: Phytoestrogens are a group of naturally occurring, non-steroidal phytochemicals. Characterized by many hydroxyl-substituted aromatic rings, these compounds have a structural similarity to endogenous estrogens, permitting them to interact with estrogen receptors and evoke estrogen-mimicking or antagonistic biological responses [58]. They are mixtures that match the structure of estrogens. In menopausal women, estrogen levels are low, so a diet abundant in phytoestrogens can exert estrogenic actions. A decreased estrogen level in menopause is a situation for neuroendocrine stimulation of hot flashes. So, an elevated intake of a phytoestrogenic diet can decrease the features of menopause [59]. Table 1 summarizes the non-hormonal modalities used for the management of menopausal vasomotor features, including lifestyle modifications and behavioral interventions, herbal and dietary options, psychotherapeutic options, and pharmacological methods.

**Table 1:** Non-hormonal options for treatment of vasomotor symptoms in menopause

Option of treatment	Category	Mechanism of action
Lifestyle modifications and behavioral interventions	Weight reduction	Reduction of body fat
	Acupuncture	Reduction of insomnia
	Systemic and organized training plan	Tranquility and controlled breathing
Herbal and dietary options	Cannabis, soy products, black cohosh, ammonium succinate, rhubarb, and probiotics and vitamin E	Controlling inflammatory, hormonal, and metabolic processes
Psychotherapeutic options	1. Cognitive behavioral therapy	Enhances adaptive mechanisms for treating vasomotor features, temper fluctuations, and sleep difficulties
	2. Mind–body training	Encourages moderate gains for both somatic and psychological well-being
Pharmacological methods	1. Selective serotonin reuptake inhibitors	Increases serotonin levels
	2. Gabapentin and pregabalin	Links to calcium channels situated in the hypothalamus and improves thermoregulatory action
	3. Antihypertensive alpha-adrenergic agonist drug (clonidine)	Reduces norepinephrine level
	4. Oxybutynin	Anticholinergic effect, so reduces sweating
	5. Neurokinin 3 receptor antagonist fezolinetant	Reduces neurotransmission by provoking neurokinin B linking on the kisspeptin, neurokinin B, and dynorphin neurons
	6. Phytoestrogen	Interacts with estrogen receptor and evokes estrogen-mimicking response

Limitations and future direction

Previous research, studies, and clinical trials on this subject need to be further reviewed. We recommend further studies and clinical trials on all non-hormonal options for treating vasomotor symptoms and the development of new options for managing these symptoms with fewer side effects. We suggest further research to directly target the thermoregulatory center involved in regulating these vasomotor symptoms.

## Conclusions

Vasomotor symptoms are one of the distressing and annoying symptoms of menopause, and finding relatively safe and effective treatment with fewer side effects and contraindications for relieving these symptoms is important. Contraindications and concerns about unwanted results with long-term hormonal treatment may account for its limited use.

Non-drug therapies comprise behavioral treatments, dietary supplements, black cohosh, selective serotonin reuptake inhibitors, gabapentin and pregabalin, clonidine, oxybutynin, and fezolinetant, which are non-hormonal treatment modalities with fewer and less serious adverse effects, complications, and contraindications than hormonal treatment.
